# Patterns of Human Respiratory Viruses and Lack of MERS-Coronavirus in Patients with Acute Upper Respiratory Tract Infections in Southwestern Province of Saudi Arabia

**DOI:** 10.1155/2017/4247853

**Published:** 2017-02-27

**Authors:** Ahmed A. Abdulhaq, Vinod Kumar Basode, Anwar M. Hashem, Ahmed S. Alshrari, Nassrin A. Badroon, Ahmed M. Hassan, Tagreed L. Alsubhi, Yahia Solan, Saleh Ejeeli, Esam I. Azhar

**Affiliations:** ^1^Unit of Medical Microbiology, Department of Medical Laboratory Technology, College of Applied Medical Science, Jazan University, Jazan, Saudi Arabia; ^2^Deanship of Scientific Affairs and Research, Jazan University, Jazan, Saudi Arabia; ^3^Special Infectious Agents Unit, King Fahd Medical Research Center, King Abdulaziz University, Jeddah, Saudi Arabia; ^4^Department of Medical Microbiology and Parasitology, Faculty of Medicine, King Abdulaziz University, Jeddah, Saudi Arabia; ^5^Department of Basic Health Sciences, Faculty of Pharmacy, Northern Border University, Arar, Saudi Arabia; ^6^Department of Public Health, Ministry of Health, Jazan, Saudi Arabia; ^7^Department of Medical Laboratory Technology, Faculty of Applied Medical Sciences, King Abdulaziz University, Jeddah, Saudi Arabia

## Abstract

We undertook enhanced surveillance of those presenting with respiratory symptoms at five healthcare centers by testing all symptomatic outpatients between November 2013 and January 2014 (winter time). Nasal swabs were collected from 182 patients and screened for MERS-CoV as well as other respiratory viruses using RT-PCR and multiplex microarray. A total of 75 (41.2%) of these patients had positive viral infection. MERS-CoV was not detected in any of the samples. Human rhinovirus (hRV) was the most detected pathogen (40.9%) followed by non-MERS-CoV human coronaviruses (19.3%), influenza (Flu) viruses (15.9%), and human respiratory syncytial virus (hRSV) (13.6%). Viruses differed markedly depending on age in which hRV, Flu A, and hCoV-OC43 were more prevalent in adults and RSV, hCoV-HKU1, and hCoV-NL63 were mostly restricted to children under the age of 15. Moreover, coinfection was not uncommon in this study, in which 17.3% of the infected patients had dual infections due to several combinations of viruses. Dual infections decreased with age and completely disappeared in people older than 45 years. Our study confirms that MERS-CoV is not common in the southwestern region of Saudi Arabia and shows high diversity and prevalence of other common respiratory viruses. This study also highlights the importance and contribution of enhanced surveillance systems for better infection control.

## 1. Introduction

Acute respiratory tract infections (ARTIs) represent a major and global cause of morbidity and mortality amongst people of all ages. Millions of children under the age of 5 die annually due to respiratory infections [1, 2]. While respiratory viruses represent a major cause of ARTIs, their prevalence and transmission are often influenced by several geographic, demographic, and environmental factors [3]. Moreover, the lack of easy and cheap diagnostic methods, the nonspecific symptoms, the large number of associated viruses, and the possibility of mixed infections usually hinder the timely and accurate identification of causative viral agents. Nonetheless, the most commonly detected human respiratory viruses are human respiratory syncytial virus (hRSV), influenza viruses (Flu), human rhinovirus (hRV), enterovirus (EV), human coronavirus (hCoV), human parainfluenza viruses (hPIV), human adenoviruses (hAdv), and human metapneumoviruses (hMPV) [4]. Several other emerging respiratory viruses such as the Middle East respiratory syndrome coronavirus (MERS-CoV) and avian influenza viruses (H5N1and H7N9) have also been reported in several other parts of the world.

In Saudi Arabia, more than 5 million cases of ARTIs are being reported annually [5] but their etiology remains largely uncharacterized due to several reasons. First, most previous studies have mainly focused on the annual Hajj pilgrimage [6–9] which might not accurately reflect locally circulating viral species. Second, while several other studies have investigated viral prevalence in ARTIs within Saudi Arabia, most of these reports have focused on specific viral pathogens [10–13]. Third, except for the city of Riyadh, the capital of Saudi Arabia [5, 11, 13–15], there is a very limited number of reports from other regions of the country [12, 16, 17].

Furthermore, the recent emergence of MERS-CoV in Saudi Arabia, its continued spread, and the associated high mortality rates (35–40%) clearly represent a serious public health and economic concern locally and globally [18]. MERS-CoV is a lineage C betacoronavirus (betaCoVs) [19] which causes symptoms ranging from asymptomatic or mild upper respiratory tract infection to severe infections associated with acute pneumonia and occasional systemic infection and multiorgan failure [20]. It was first reported in Saudi Arabia in 2012 [21] and later from 26 countries in the Arabian Peninsula, Africa, Europe, Asia, and North America [22]. Almost all reported MERS-CoV cases were linked to the Arabian Peninsula. Frequent sporadic cases of MERS-CoV and multiple hospital outbreaks have been reported in several cities in Saudi Arabia including Alahsa, Taif, Jeddah, and Riyadh [23, 24].

Jazan province is located in the southwestern part of Saudi Arabia just north of Yemen. It is the second smallest and the most densely populated province in Saudi Arabia with density of ~132/km^2^ [25]. So far, there is barley any report on the prevalence of respiratory viruses in this region of the country. Furthermore, while several MERS-CoV surveillance studies have been conducted in several parts of Saudi Arabia, only few reports have examined the seroprevalence of MERS-CoV in Jazan province and found no serological evidence of MERS-CoV circulation in this region [26, 27]. These data are consistent with the overall prevalence pattern of MERS-CoV in Saudi Arabia especially that only one single MERS case has been reported from this province since 2012 [28]. Therefore, we undertook enhanced surveillance of those presenting with respiratory symptoms at five healthcare centers by testing all symptomatic outpatients between November 2013 and January 2014 (winter time) in southwestern region of Saudi Arabia.

## 2. Material and Methods

### 2.1. Samples

A total of 182 samples were collected from five healthcare centers in Jazan province, Saudi Arabia, between November 2013 and January 2014 (winter time). Nasopharyngeal swabs were collected from all symptomatic outpatients at all ages who presented with symptoms of ARTI to these five healthcare centers. Swabs were collected from 131 male and 51 female patients. All swabs were collected in virus transport media, stored at −80°C, and transported for processing at the Special Infectious Agents Unit, King Fahd Medical Research Center, King Abdulaziz University, Jeddah, Saudi Arabia. Ethical approval was obtained from Jazan University Ethical Committee (JUEC). All clinical information and laboratory results were collected and informed consent was obtained from all adult patients and parents of children.

### 2.2. RNA Extraction and cDNA Synthesis

In order to control for RNA extraction, samples were thawed on ice and 2 *µ*l of armored RNA (aRNA) of hepatitis C virus (genotype 1a) was added to 200 *µ*l of each sample as an internal control. RNA extraction was then carried out using QIAamp Viral RNA mini kit (Qiagen, Germany) according to manufacturer's instructions with final elution volume of 40 *µ*l, and extracted RNA was stored at −80°C until use. Viral RNA was then reverse-transcribed using SuperScript III First-Strand Synthesis SuperMix kit (Invitrogen, USA). The reaction mixture consisted of 1 *µ*l of random primers, 1 *µ*l annealing buffer, and 6 *µ*l of extracted RNA containing aRNA in a total volume of 8 *µ*l. The mixture was incubated at 70°C for 5 min, and then the PCR block was cooled to 4°C. After that, 10 *µ*l of 2x first-strand reaction buffer and 2 *µ*l of enzyme mix were added to the mixture and incubated at 25°C for 5 min and then at 50°C for 50 min. Reaction mixture was then incubated at 85°C for 5 min to inactivate enzymes and cDNA was stored at −80°C until use. All samples were tested with Infiniti RVP plus assay on Infiniti Plus analyzer which identifies the following respiratory viruses: Influenza A virus (FluA), Influenza B virus (FluB), Influenza A-Swine H1N1 virus, hPIV (1, 2, 3, and 4), hRV (A and B), EV (A, B, C, and D), hCoV (HKU1, OC43, NL63, and 229E), hMPV (A and B), hRSV (A and B), and hAdv (A, B, C, and D) with 90% sensitivity and 100 specificity according to manufacturer (AutoGenomics, USA).

### 2.3. RVP Plus Infiniti Microarray Assay

Generated cDNA was PCR amplified in a multiplex reaction by adding 9.9 *µ*l of ready to use amplification mixture containing dNTPs, multiplex primers mix, MgCl2 and reaction buffer (AutoGenomics, USA), 0.1 *µ*l of platinum Taq polymerase (Invitrogen, USA), and 10 *µ*l of cDNA. Cycling conditions were performed as follows; one cycle at 94°C for 2 min, followed by 39 cycles consisting of denaturation at 94°C for 30 sec, annealing at 55°C, and extension at 72°C for 1 min. Reaction was ended with a final extension at 72°C for 3 min followed by hold at 4°C. PCR products were then cleaned up from remaining dNTPs and PCR primers by adding 3 *µ*l of Shrimp Alkaline Phosphatase (SAP), 0.75 *µ*l of Exonuclease I (EXO) (GE Life Sciences, USA), and 0.5 *µ*l of titanium Taq polymerase (Invitrogen, USA) to each PCR product, and the mixture was incubated for 30 min at 37°C, followed by 10 min at 94°C and a final hold at 4°C. All samples were then loaded in the Infiniti Plus analyzer for testing with RVP plus assay (AutoGenomics, USA) according to manufacturer's instructions. Briefly, samples were subjected to primer extension reaction using detection primers (AutoGenomics, USA) and fluorescent labeling with fluorescent nucleotides of the amplified product, followed by hybridization of the tagged amplified products to corresponding probes on the DNA microarray chips. Chips were then washed, scanned, and signals were recorded and analyzed. Specimen was considered positive when the ratio between virus signal and background signal was above threshold calculated by the manufacturer's software.

### 2.4. MERS-CoV RT-PCR

Extracted RNA from all samples was subjected to MERS-CoV upstream E-gene (UpE) detection using real-time RT-PCR on LightCycler 2.0 (Roche, Germany) as previously described [29] in a final volume of 20 *µ*l. Negative (no-template control (NTC)) and positive controls were always included.

### 2.5. Statistical Methods

Data were statistically analyzed using the Statistical Package for Social Science software (SPSS v20.0; IBM Crop, Armonk, NY, USA). Descriptive statistics for continuous variables were compared using the nonparametric Mann–Whitney *U* test or the Kruskal-Wallis test. For categorical variables, the *χ*^2^ test, the Fisher exact test, or the *Z* test was applied to evaluate the difference between proportions or to assess whether there were any associations between the proportions. A two-tailed probability value *p* < 0.05 was considered statistically significant. Values were expressed as mean or standard deviation and percentages wherever necessary.

## 3. Results

### 3.1. Epidemiological Data

Of the samples tested, 41.2% (75/182) were positive for one or more viruses (Table 1) including 72% (54/75) males and 28% (21/75) females. Saudis accounted for 84% (63/75) whereas non-Saudis represented 16% (12/75) of all the positive cases. Prevalence of positive samples was similar regardless of gender or ethnicity. Specifically, detection rates were 41.2% (54/131) and 41.1% (21/51) amongst all males and female patients, respectively, and 40.7% (63/155) and 44.44% (12/27) in Saudis and non-Saudis, respectively. Similarly, smoking seems to have no significant effect on the detection of respiratory viruses as rates were 39.4% (13/33) and 41.61% (62/149) in smokers and nonsmokers, respectively. As shown in Table 1, presentation to healthcare centers and detection rate of respiratory viruses gradually decreased with age. Respiratory viruses were more commonly detected in individuals under the age of 15 years who represented 34.7% (26/75) of the infected patients. Nonetheless, viral detection rate was 61.9% (13/21) in patients who aged 35–44 and presented to healthcare centers. This was followed by those who were older than 44 (45.45%, 5/11) and individuals under the age of 15 (41.94%, 26/62), indicating that presentation to healthcare facilities by older adults, compared to children under the age of 15 years, is usually associated with actual infections. Interestingly, marked differences in detection rates were observed between the different healthcare centers within the region. As shown in Table 1, viruses were more commonly detected in healthcare center 3 (48%, 36/75) and healthcare center number 4 (29.3%, 22/75) compared to the remaining 3 centers.

### 3.2. Viral Prevalence and Clinical Profile

All the samples collected in this study were first tested for MERS-CoV by RT-PCR. Consistent with the current prevalence and circulation pattern of MERS-CoV in Saudi Arabia, all samples were deemed negative for MERS-CoV, suggesting that there is a lack of nasal carriage of MERS-CoV in the southwest region of Saudi Arabia. Next, all samples were tested for a variety of respiratory viruses using Infiniti RVP plus microarray assay. As shown in Table 2, out of the 75 infected patients, 62 (82.7%) were infected with a single respiratory virus. On the other hand, 17.3% (13/75) had coinfections with two respiratory viruses.

The most frequently detected viruses were hRV (40.9%, 36/88), hCoV-OC43 (15.9%, 14/88), FluA (13.6%, 12/88), hRSV-B (10.2%, 9/88), and hAdv (5.7%, 5/88). The most frequent coinfecting virus was hRV which was detected in 7 out of the 13 coinfected patients representing 26.9% (7/26) of the coinfected viruses (Table 2). This was followed by hCoV-OC43 (19.2%, 5/26), FluA (19.2%, 5/26), hAdv (15.4%, 4/26), hRSV-A (7.7%, 2/26), hRSV-B (7.7%, 2/26), and EV (3.9%, 1/26). Human RV was most commonly detected with hAdv (4/7) which was the most common coinfection (30.8%, 4/13) followed by hCoV-OC43 and FluA coinfection (23.1%, 3/13). The remaining 6 coinfections were detected once and were due to unique combination of respiratory viruses (Table 3). Human RV was the only virus that was detected in all age groups and most commonly in the age group of 25–34 compared to younger or older individuals (Table 3). Interestingly, while male patients had more coinfections than females, detection of more than one virus decreased with age and completely disappeared in people older than 45 years regardless of gender (Table 3).

When comparing the overall clinical symptoms of infected and noninfected patients (Table 4), we found a significant association between the positive detection of viral infections and presentations of runny nose (*p* = 0.014), wheezing (*p* = 0.007), or lethargy (*p* = 0.035) to healthcare facilities. While the small number of patients limited our ability to examine the association between each viral infection and clinical symptoms, we observed a significant association between nasal congestion and single infections (*p* = 0.0163) compared to dual infections (Table 5).

## 4. Discussion

In the present study, we screened clinical samples collected from 182 symptomatic patients that presented with suspected ARTIs in the southwestern province of Saudi Arabia not only for MERS-CoV but also for other respiratory viruses. While several respiratory viruses were detected in 41.2% of the patients, we could not detect any evidence of MERS-CoV in the study population which is in accordance with previous reports from the region [26, 27]. Noteworthy, Alagaili and colleagues studied geographical circulation of MERS-CoV in dromedary camels in the Kingdom of Saudi Arabia in 2013 and did not find any evidence of MERS-CoV in Jazan province [30].

**Figure 1:**
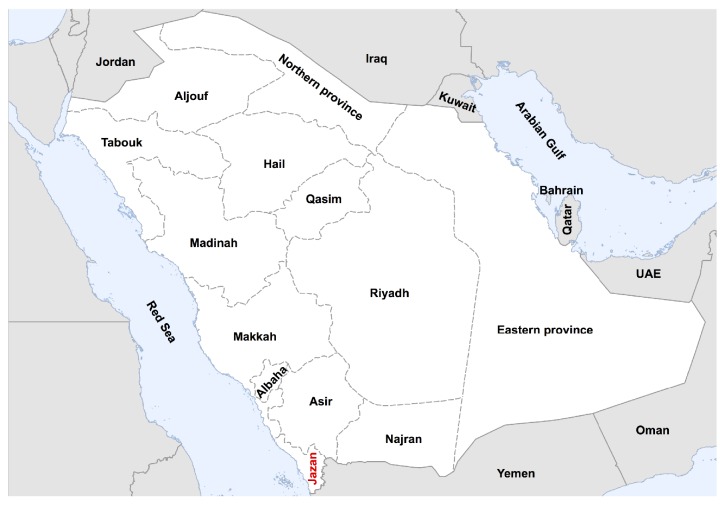
Map of Saudi Arabia showing the administrative provinces. Jazan province (red) is in the southwestern region of the country and north of Yemen.

Interestingly, viral prevalence was almost similar in children under the age of 15 years and individuals older than 15 years. Specifically, 26 out of 62 children (41.9%) tested positive for one or more viruses. Similarly, 40.1% of all adults who are older than 15 years were infected. The rate of infection in children in our study is lower than previously reported rate in Riyadh (~61%) [13] or in Najran province in the southern region of Saudi Arabia (>74%) [17] (Figure 1). However, this difference is expected as both of these previous studies have mainly focused on hospitalized children ≤5 years of age. In contrast, we screened outpatients in primary healthcare settings and used a wider age range which is also consistent with the decrease in detection of respiratory viruses with increasing age (Table 1). Nonetheless, children in our study represented the largest proportion (34.7%) of all infected patients compared to other age groups.

Human RV accounted for more than 40.9% of the pathogens identified, followed by non-MERS-CoV human coronaviruses (19.3%), Flu viruses (15.9%), and hRSV (13.6%). On the other hand, detection of other respiratory viruses (hAdv, EV, and hMPVA) comprised 10.2% of all identified viruses. While hRV, Flu A, and hCoV-OC43 were more prevalent in adults older than 15 years, they were detectable in patients from most age groups. In contrast, RSV, hCoV-HKU1, and hCoV-NL63 were mostly restricted to children. Our data clearly suggest that hRV is an important cause of ARTIs in addition to Flu A and hCoV-OC43 during winter season. The overall prevalence of respiratory viruses in this study is in accordance with previous reports of respiratory viruses in children [5, 13, 17] and adults [9, 31] from Saudi Arabia. However, our data showed lower levels of RSV and hMPVA in children compared to studies from Yemen which is in very close proximity to Jazan province [32, 33]. While further studies are clearly required, these differences could be due to the additional risk factors in Yemen especially in children [32, 33].

Coinfection was not uncommon in this study, in which 17.3% of the infected patients had dual infection consistent with a recent study from Turkey [34]. However, it is higher than previously reported rates in children from Saudi Arabia [5, 17] most likely due to the methodological differences between the studies. The most common coinfecting virus was hRV (7/13) and it was detected with most viruses including hAdv, FluA, hRSV, and hCoV-OC43. Other frequently coinfecting viruses were FluA, hCoV-OC43, hRSV, and hAdv. Notably, hCoV-OC43 was the only coinfecting coronaviruses amongst the other non-MERS-CoV coronaviruses. Interestingly, hAdv was more frequently detected in coinfections (4 times) compared to single infections (one time), and all these coinfections were with hRV only. Furthermore, dual infections were more common in children and young adults between 15 and 24 years compared to other age groups. While viral coinfections are very frequent in hospitalized children with ARTIs, the impact of such infections in outpatients is not clear especially that several studies concluded that coinfections may or may not contribute to increased disease severity and the risk for hospitalization [35–38]. Nonetheless, more studies are required to better understand the clinical impact of coinfections.

Interestingly, the detection of respiratory viruses varied significantly between regions in our study. Two centers (3 and 4) provided more than 77% of the positive cases in the current study, suggesting that these centers may serve a large community in the region compared to the other 3 centers and thus they could represent suitable sentinels for future surveillance studies. The similarity in clinical symptoms and manifestations of patients infected not only by respiratory viruses but also by some bacterial agents represent a hurdle in diagnosis based on clinical presentation. Although we observed some association between viral infection and some clinical symptoms such as running nose, lethargy, and wheezing, the overall array of symptoms were not specific most probably due to the small size of samples in this study. Other limitations in our study included the short studied period (winter season) and focusing of viral agents only. Nonetheless, the rapid advancements in molecular diagnostic methods such as microarray or multiplex PCR should aid in the epidemiological characterization of circulating viruses.

In conclusion, our data shows that circulating viruses in the southwestern province of Saudi Arabia are highly diverse and hRV represent a major pathogen in all age groups during winter season. Furthermore, it shows that MERS-CoV is infrequent in this region of Saudi Arabia compared to other regions most probably due to the limited number of dromedary camels, the reservoir host for MERS-CoV, and consequently their direct contact. Finally, use of multiplex assays such as the one used herein could help in the determination of the spectrum and diversity of respiratory viruses and in the implementation of effective control measures by public health authorities. This work shows the importance of enhanced surveillance in understanding the epidemiology of respiratory infections and therefore applying the appropriate control measures.

## Figures and Tables

**Table 1:** Summary of demography characteristics of patients with upper respiratory tract infection in the community in Jazan province, Saudi Arabia.

Variable	Infected	All subjects
	Number (%)	Number (%)
Total number	75 (41.2)	182 (100)
Gender		
Male	54 (72.0)	131 (72.0)
Female	21 (28.0)	51 (28.0)
Nationality		
Saudi	63 (84.0)	155 (85.2)
Non-Saudi	12 (16.0)	27 (14.8)
Smoking		
Yes	13 (17.3)	33 (18.1)
No	62 (82.7)	149 (81.9)
Age group (years)		
<15	26 (34.7)	62 (34.1)
15–24	16 (21.3)	48 (26.4)
25–34	15 (20.0)	40 (22.0)
35–44	13 (17.3)	21 (11.5)
>45	5 (6.7)	11 (6.0)
Healthcare center^¶^		
Center 1	2 (2.7)	2 (1.1)
Center 2	10 (13.3)	19 (10.4)
Center 3	36 (48.0)	68 (37.4)
Center 4	22 (29.3)	87 (47.8)
Center 5	5 (6.7)	6 (3.3)

^¶^Center 1 (Al Rowdah District, North), Center 2 (Al Rowdah District, South), Center 3 (Area 5), Center 4 (Al Safa District), and Center 5 (Al Shatea District).

**Table 2:** Viruses identified in patients with upper respiratory tract infection.

Viruses	Single infections (*n* = 62)	Coinfections (*n* = 26)^¶^	All infections (*n* = 88)^$^
	Number (%)	Number (%)	Number (%)
hRV	29 (46.8)	7 (26.9)	36 (40.9)
hCoV-OC43	9 (14.5)	5 (19.2)	14 (15.9)
hCoV-HKU1	2 (3.2)	0	2 (2.3)
hCoV-NL63	1 (1.6)	0	1 (1.1)
hRSV-A	1 (1.6)	2 (7.7)	3 (3.4)
hRSV-B	7 (11.3)	2 (7.7)	9 (10.2)
FluA	7 (11.3)	5 (19.2)	12 (13.6)
FluB	2 (3.2)	0 (0.0)	2 (2.3)
EV	2 (3.2)	1 (3.9)	3 (3.4)
hAdv	1 (1.6)	4 (15.4)	5 (5.7)
hMPVA	1 (1.6)	0	1 (1.1)

^¶^Coinfections include all viruses detected from the 13 coinfected patients (2 × 13).

^$^Includes viruses detected in single infections (62) and in coinfections (26).

**Table 3:** Demographic data of patients with upper respiratory tract infection by pathogen.

Infecting viruses	Number of patients	Age (years)					Gender	
		Number (%)					Number (%)	
		<15	15–24	25–34	35–44	>45	Male	Female
Total number	75	26 (34.7)	16 (21.3)	15 (20.0)	13 (17.3)	5 (6.7)	54 (72.0)	21 (28.0)
hRV	29	5 (19.2)	6 (37.5)	9 (60.0)	5 (38.5)	4 (80.0)	24 (82.8)	5 (17.2)
hCoV-OC43	9	3 (11.5)	1 (6.3)	1 (6.66)	4 (30.8)	0	7 (77.8)	2 (22.2)
hCoV-HKU1	2	1 (3.8)	0	0	0	1 (20.0)	0	2 (100)
hCoV-NL63	1	1 (3.8)	0	0	0	0	1 (100)	0
hRSV-A	1	0	1 (6.3)	0	0	0	1 (100)	0
hRSV-B	7	5 (19.2)	2 (12.5)	0	0	0	4 (57.1)	3 (42.9)
FluA	7	2 (7.7)	1 (6.3)	2 (13.33)	2 (15.4)	0	5 (71.4)	2 (28.6)
FluB	2	1 (3.8)	0	1 (6.66)	0	0	1 (50)	1 (50)
EV	2	2 (7.7)	0	0	0	0	1 (50)	1 (50)
hAdv	1	0	1 (6.3)	0	0	0	1 (100)	0
hMPVA	1	0	0	1 (6.66)	0	0	1 (100)	0
hRV + FluA	1	0	1 (6.3)	0	0	0	1 (100)	0
hAdv + hRV	4	2 (7.7)	1 (6.3)	1 (6.66)	0	0	2 (50)	2 (50)
hCoV-OC43 + FluA	3	1 (3.8)	1 (6.3)	0	1 (7.7)	0	3 (100)	0
hRV + hRSV-A	1	1 (3.8)	0	0	0	0	1 (100)	0
hRSV-A + hRSV-B	1	1 (3.8)	0	0	0	0	0	1 (100)
hCoV-OC43 + hRSV-B	1	0	1 (6.3)	0	0	0	1 (100)	0
FluA + EV	1	1 (3.8)	0	0	0	0	0	1
hCoV-OC43 + hRV	1	0	0	0	1 (7.7)	0	0	1

**Table 4:** Clinical symptoms in infected and noninfected patients.

Clinical symptoms	Infected (*n* = 75)	Non-infected (*n* = 107)
	Number (%)	Number (%)
Fever	54 (72)	65 (60.7)
Cough	68 (90.7)	96 (89.7)
Runny nose	67 (89.3)^*∗*^	80 (74.8)
Wheezing	17 (22.7)^*∗*^	9 (8.4)
Headache	36 (48.0)	38 (35.5)
Sore throat	62 (82.7)	88 (82.2)
Difficult breathing	8 (10.7)	11 (10.3)
Lethargy	30 (40.0)^*∗*^	27 (25.2)
Nausea	10 (13.3)	10 (9.3)
Nasal congestion	59 (78.6)	72 (67.3)
Earache	7 (9.3)	12 (11.2)

^*∗*^Significant *p* value < 0.05.

**Table 5:** Clinical symptoms in patients with single infections and coinfections.

Clinical symptoms	Single infection (*n* = 62)	Coinfection (*n* = 13)
	Number (%)	Number (%)
Fever	43 (69.4)	11 (84.6)
Cough	55 (88.7)	13 (100)
Runny nose	56 (90.3)	11 (84.6)
Wheezing	13 (21)	4 (30.8)
Headache	29 (46.8)	7 (53.8)
Sore throat	51 (82.3)	11 (84.6)
Difficult breathing	5 (8.1)	3 (23.1)
Lethargy	25 (40.3)	5 (38.5)
Nausea	8 (12.9)	2 (15.4)
Nasal congestion	52 (83.9)^*∗*^	7 (53.8)
Earache	4 (6.5)	3 (23.1)

^*∗*^Significant *p* value < 0.05.
